# New Horizon in Life: Experiences of Patients Receiving Chemotherapy

**DOI:** 10.5539/gjhs.v8n4p102

**Published:** 2015-07-30

**Authors:** Alireza Nikbakht Nasrabadi, Ali Mohammadpour, Mohammad Fathi

**Affiliations:** 1 School of Nursing and Midwifery, Tehran University of Medical Sciences, Tehran, Iran; 2 Gonabad University of Medical Sciences Gonabad, Iran; 3 School of Nursing and Midwifery, Tehran University of Medical Sciences, Tehran, Iran

**Keywords:** chemotherapy, cancer, qualitative research

## Abstract

**Introduction:**

The treatment quality of diseases can affect the patient's experience. Due to its different complications among cancer patients, the experience of chemotherapy is unique. The present study was conducted to explore the lived experience among cancer patients who had received chemotherapy.

**Methods:**

The study was conducted by a qualitative approach and a phenomenological method. In so doing, 12 cancer patients who had received chemotherapy were purposefully selected were interviewed using an in-depth method. After the required data were collected, they were analyzed by Tanner, Allen, Diekelmann method.

**Results:**

Analysis of the collected data indicated that the experience of chemotherapy appeared as “a new horizon in life” for the patients. Secondary themes of the new horizon in life included rebirth, understanding of life values, dependence, and need.

**Conclusion:**

According to the results of the study, it was concluded that in addition to taking into providing mental-spiritual support and reducing the complications of the treatment, nurses in chemotherapy wards should pay attention to the experiences of the patients receiving chemotherapy and enhance hope and positive attitude among them.

## 1. Introduction

Cancer is the fourth cause of mortality all over the world and the most important health problem in Middle Eastern countries (Omar et al., 2007). In Iran, cancer is the third cause of mortality (Radmard, 2010). According to the official annual statistics, about 70,000 new cases of cancer emerge and more than 30,000 people die of this disease every year (Mousavi et al., 2009). Chemotherapy is considered as the main and a systematic medical intervention (American Cancer Society, 2013). Such that over 50% of the cancer patients receive chemotherapy (Ignoffo and al., 2008).

Despite of various applications, chemotherapy is involved with different side complications for and harsh effects on the patient's general health in emotional, social, physical, and spiritual dimensions (Roffe et al., 2005). Chemotherapy basically creates an important crisis in all different dimensions of the patient's life, such that some patients have declared chemotherapy is worse that the cancer itself (Baker and Ellett 2007).

Nowadays, the most important role of the nurses as the professional forces comprising the health and medical system is to carry out rudimentary care (Newell et al., 2002). Nurses are main responsible practitioners that carry out chemotherapy and provide patients with extensive care; therefore, they should be familiar with all aspects of this important medical phenomenon from the patient's perspective (Gibson and et al., 2013; Kralik et al., 2001, McGrath, 2001). There is no doubt that taking into consideration the phenomenon of chemotherapy and its effects on the patient's physical and mental aspects and different individual dimensions of life, family, and society is among important measures that can enhance the patient's life quality. Examining, knowing, and taking action in this regard are more expected from nurses than any other members of medical teams (Clarke et al., 2002). Discovering these patients’ experiences and examining their emotional, spiritual, mental, physical, and even family and social issues and problems can greatly help with providing realistic care programs that are based on real concepts of this experience (Gibson and et al., 2013; Kralik et al., 2001; McGrath, 2001). Acquiring knowledge about chemotherapy experience has a unique significance for nursing students and nurses and provides an appropriate opportunity for other studies to be conducted in this field. Awareness of social-cultural issues, beliefs, strong religious values, and all other issues related to this phenomenon are significant (Nikbakht Nasrabadi et al., 2011).

Culture causes the individuals to interpret diseases, suffrage, and death. Therefore, understanding the lived experience of the individuals that are receiving chemotherapy by the professional staff of medical team is necessary in order to provide appropriate information and figure out related and acceptable treatments (Doumit et al., 2007). Based on the definition proposed by WHO, the individuals’ experience of their own situation is related to their cultural background, the value systems that they are living in, and their goals, expectations, and standards (Higginson & Robinson, 2002). In total, attempts to get familiar with the individuals’ experiences is important due to deep effects of chemotherapy on patients and their families and in order to provide appropriate care and support the patients and their families because successful management of the patients and their family caretakers requires an extensive understanding of their experiences (Doumit et al., 2007). In fact, since there was not a clear definition for the lived experience of the cancer patients receiving chemotherapy in educational and clinical environments of Iran, qualitative methodology seemed to be an appropriate method to conduct the present study because this method makes it possible to reach out for the participants’ internal experiences and discover related variables through in-depth exploration of phenomena. There are various methodologies such a phenomenology. From a phenomenological perspective, mental interpretation is an inevitable matter. Moreover, the study of man and the world is a hermeneutic science and seeks meaning which forms within the individuals’ everyday interactions and relationships. A phenomenologist's ultimate goal is to clarify the nature, meaning, context, and quality of the participants’ experiences of an issue (Holloway & Wheeler, 2010). Therefore, according to unique complexities of man as an important phenomenon in the field of health and the most important meta-paradigm in the field of nursing, due to the point that few studies have been able to consider different aspects of human life in-depth especially while he is receiving chemotherapy, considering differences in Iranians’ living conditions, beliefs, deeds, and culture compared to other countries, and since understanding the experiences of such patients adds to nursing knowledge, the present study was conducted on a group of patients under chemotherapy using a qualitative phenomenological method.

## 2. Methods

Since the present study was aimed at exploring the experiences of the patients receiving chemotherapy, phenomenological method was employed. Phenomenology is an approach to philosophy and nowadays a method of inquiry. Various way of doing Phenomenology exist. They all have similar aims. The major aim of phenomenological approach is to generate a description of a phenomenon of everyday experience to achieve an understanding of its essential structure (Holloway & Wheeler, 2010).

### 2.1 Participants

The participants were selected in a purposeful method. They were patients who had received at least once or had finished the treatment. Inclusion criteria were the age of 18 and over it, tendency to participate in the study, and willingness to provide their experiences. They were identified by considering their medical profile and consulting with their doctor, the head nurse, the ward personnel, and other patients. The study data were generally collected Oncology and Chemotherapy Wards of Sanandaj's Tohid Hospital. Fourteen patients participated in the study. The process of selecting the participants continued until a rich interpretation was achieved and no new data appeared during the interviews.

### 2.2 Data Collection

We employed the personal in-depth semi-structured interview method for collecting the data. All interviews were arranged according to participants’ convenience and were conducted either in a quiet room located in the study setting or at the first author's office in Sanandaj Faculty of Nursing and Midwifery. Each interview was started by asking a general question, ‘Would you please tell me more about your experiences of chemotherapy?’ Then, questions such as ‘What does chemotherapy mean to you?’ and ‘Chemotherapy is like…?’ were used for acquiring more comprehensive understanding of participants’ experiences. Totally, fourteen interviews were conducted with nine participants. Interviews lasted for 35–90 minutes. We tape-recorded the interviews and saved them as computer files.

### 2.3 Data Analysis

Study data were analyzed by employing the seven-step approach proposed by Diekelmann, Allen, and Tanner (1989). This hermeneutic approach is commonly used for qualitative data analysis and helps achieve the highest possible level of abstraction (Gibson and et al., 2013). Initially, we carefully read each interview transcript to gain an overall understanding of it (step 1). An interpretive summary was written for each interview (step 2). The research team discussed and negotiated with each other about the generated themes. Each member of the team freely shared his/her idea about the findings (step 3). Then, ambiguities and disagreements were clarified and resolved by referring to either the transcripts or the participants (step 4). After identifying, clarifying, and resolving ambiguities and disagreements, interpretive summaries were revised and rewritten. Final interpretive summaries were assessed and scrutinized again by the research team. After reaching a general agreement on the revised/rewritten interpretive summaries based on the union of researchers and participants’ horizons, the basic pattern of the common meanings and shared practices were identified (steps 5). Thereafter, we strived to identify the relationship(s) among the generated themes (step 6). A draft of the themes in addition to selected excerpts from the interviews were presented to the interpretive team. Their suggestions were incorporated into the final draft (step 7). Data collection and analysis were performed in 2014.

The Lincoln and Guba's four-criterion gold standard—consisting of credibility, dependability, conformability, and transferability—was used for maintaining the rigor of the study. The credibility of the findings was maintained by keeping a prolonged engagement with the data (more than nine months), referring to clinical settings and establishing effective communication with participants, and employing the member- and peer-checking techniques (Speziale et al., 2011). The main focus of the Diekelmann's approach is on discussing, negotiating, and arriving at a general agreement on the data and the findings. To establish the dependability and confirmability of the findings, we clearly explained and documented the flow and the steps of the study.

### 2.4 Moral Considerations

This study was approved by Tehran Faculty of Nursing and Midwifery, Tehran, Iran, in January 15, 2014 (approval code: 92/D/130/2397). We took into accounts all research-related ethical considerations including receiving approval from ethics committee, obtaining informed consent from the participants, obtaining participants’ permission for recording the interviews, clearly explaining the aims of the study, maintaining their anonymity, and preserving their right for withdrawing from the study. During data analysis and also in the final report of the study, we referred to the participants by using numerical codes. Study participants were also ensured that they would be able to have access to the findings of the study.

## 3. Results

Based on the collected data in the present study, chemotherapy was associated with rebirth, understanding of life values, dependence, and need, which finally led to a “new horizon in life” as the main theme of the study.

The participants related the gradual improvement in their different physical, spiritual, and mental aspects first to God's kindness then to chemotherapy. In other words, they understood the effectiveness of chemotherapy in their life and stated that if they had not undergone chemotherapy, they would have been in such a worse condition that they could not continue their lives; therefore, their lives had taken a new trend and their health had returned as a result of chemotherapy.

### 3.1 Rebirth

One of the secondary themes of the study was rebirth. The participants declared that after they heard about their cancer, they were filled with the fear of death; however, chemotherapy caused hope to live among them. Following statements are caused by the participants’ experiences of the positive effect of the treatment and the return of their hope of life.

One of the participants talked about his rebirth this way, “Chemotherapy is effective in removing cancer cells, so I believe that chemotherapy has a mechanism of rebirth that causes a new life to begin” [1]. Another participant said, “For me, chemotherapy is a means for survival and rebirth. Like a doll that gives a child relaxation, chemotherapy is the same for me” [11]. Another participant's experience was like this, “Now I’m highly hopeful that I get recovered. I’m in peace now. I come here excitedly. I’ve undergone chemotherapy twice and get myself ready for other sessions” [7].

### 3.2 Understanding of Life Values

Understanding the values of life was one of the secondary themes. After chemotherapy, motivations for life rose. Some participants’ experiences were as follow. “Instead of crying and losing my hope and saying that my life is finished, my hope for life has risen, and right now I enjoy every single second of my life. You know how it is like, a health person may not think about life a lot, but when one is under chemotherapy he would say he may die any time, so he needs to make use of all moment of his life. I pay more attention to positive aspects of chemotherapy and get much energy from it. Instead of recessing, my life has progressed much” [5]. Another participant's experience was like this, “I don’t say it's frightening, but it's a little tough. When you receive chemotherapy, you need to be more careful so you can lead a healthy life” [10]. Another participant refers to the same issue like this, “The first point is that you will appreciate your health more and take more care of yourself. Paying attention to your health will rise. Chemotherapy enhances the patient's tolerance. It seems that it makes you tough against difficulties” [12].

### 3.3 Dependence

While expressing their experiences, the participants referred to the issue that chemotherapy had caused them to have a better understanding of friends and fellows. In this regard, one of the participants stated, “Another experience that chemotherapy gave me is understanding my friends and fellows more. Now I look differently at issues that were not important to me before. I’m more dependent on my family and friends. I have a better understanding of my relationships with my friends” [4]. For another participant, dependence was experienced this way, “I’d like have more close relationships with my fellows and friends. Although sometimes you may not like to talk to anyone after chemotherapy, its [talking to others] causes emotional unity” [9]. Another participant's dependence was like this, “After chemotherapy, I paid more attention to people and my fellows. Previously when someone would say he is sick, I would pray for him, but since I’ve undergone chemotherapy, I understand my fellows better” [11].

### 3.4 Need

The statements made by the participants indicated that they had understood the fact that they needed chemotherapy to have their health back, and they referred to it as return to health and dependence on chemotherapy. “Chemotherapy gave me my life back, and now I’m hopeful of life and appreciate it better. I feel that Almighty God has given me peace and health through chemotherapy” [1]. Another participant stated, “I’m a patient who needs a long period of chemotherapy just like any other patient so that I can return to my normal life” [11]. Another participant also said, “I’ve noticed that chemotherapy has a surviving role for my life and is useful, and I would not be here now and couldn’t interview with you if I didn’t undergo chemotherapy” [3].

One of the aspects of the participants’ needs was their reliance on chemotherapy. “Chemotherapy is like an addiction to me. I come to Sanandaj so that my need can be satisfied. If I don’t receive chemotherapy, I’ll feel bad. Chemotherapy has become a need for me and it makes me feel better. I feel it gives me mental and physical strength” [11]. “You know what chemotherapy is like to me? It's like an infant that needs his/her mother's breast milk. The same way that an infant's needs are satisfied with the breast milk, my needs will be with chemotherapy. I see myself like an infant and a person who is hungry and needs food. I need chemotherapy so I can resolve my problems” [4].

In general, the participants’ experience of chemotherapy was interpreted as a “new horizon in life”. They felt that chemotherapy had given them a new horizon of life through rebirth, understanding of life values, dependence, and need.

## 4. Discussion and Conclusion

The results of the study indicated that a dimension of lived experience or a main theme among the cancer patients receiving chemotherapy was a “new horizon in life”. This theme included four sub-themes of rebirth, understanding of life values, dependence, and need. Most of the participants of the present study stated that they had experienced many problems during chemotherapy but after a maximum of one week or ten days they had returned to their initial state and the complications of the chemotherapy reduced. The metaphor of rebirth was generated from the data. The participants experienced the chemotherapy process as a life changing and this was expressed by most individuals as a rebirth. For some of them it was a physical rebirth as feeling physically better that they did prior to becoming ill and for some it was a psychological rebirth as feeling remaining in the family and society. As it emerged from this experience they had a new life and a new perspective; they were transformed and displayed this transformation through deeper connections to themselves and others (Lawrence and Cross, 2013). These findings were in agreement with other ones that indicated that the quality of life may drop during chemotherapy but after some time the patient's health will increase (Hawighorst-Knapstein et al., 2004). In his study, Miller (2002) concluded that patients with gynecologic cancer had a better state of health after chemotherapy; however, there was no significant difference between the patients and healthy individuals (Miller et al., 2002). In their study, Chan et al (2003) concluded that after chemotherapy the quality of life had improved and continued for 12 months. Moreover, in another investigation Chan et al (2001) studied 144 patients with newly diagnosed gynecologic cancer before and after chemotherapy and concluded that the total health state had improved completion of chemotherapy (Chan et al., 2001).

Moreover, the results of the study conducted by Tahmasebi et al (2007) showed that the mean score of life quality among patients receiving chemotherapy to treat their ovarian and cervical cancers was significantly higher than before the treatment (Tahmasebi et al., 2007). In their studies, Von Gruenigne et al. (2010) declared that patients with ovarian cancer experienced decrease in their physical, functional, and mental aspects and life quality, which improved after chemotherapy (von Gruenigen et al., 2010). Diagnosis of cancer and reception of chemotherapy are equal to passing from one stage to another and creating a new balance toward future goals and achievement of a normal life. Participants consider their lives to be dependent on chemotherapy. They think it has given their lives a certain rhythm and regulation, they have talked about chemotherapy as their small internal world against the external world. In fact, presence at hospital has become a part of their lives (Mcilfatrick et al., 2003). Culture include knowledge, belief, morals, law, custom, and any other capabilities and habits acquired by man as a number of society. Iran is an ancient country located in the Middle East with more than 5000 years of culture and a population of about 70 millions, mostly Muslim. Cancer is the third cause of death in Iran, about 100,000 people are being diagnosed with this disease annually. In Iran being diagnosed with cancer has cultural, social and psychological consequences. Many patients and their relative experience physical, psychological, spiritual and family problems. So for Iranian cancer patients spiritual values are important. Spirituality is the individual's sense of peace, purpose, and connection to others, and beliefs about the meaning of life. Therefore the spiritual component of care is important and should be considered in the care of cancer patients undergoing chemotherapy (Hemati et al., 2014).

Finally, it can shortly be stated that this group of patients’ experience of chemotherapy caused them to feel rebirth after treatment, understand the values of their lives better, come up with more dependence, and even feel more in need, which in total created a new horizon in their lives.

Although in the present study there were lots of considerations to enhance the study's precision, its generalizability was limited due to its qualitative approach. Therefore, further studies are needed in order to explore the experiences of the patients receiving chemotherapy. Moreover, further studies are required to explore the way the experiences of certain cancers are analyzed.

## 5. Conclusion

In general, the patients had a positive attitude toward chemotherapy and created a good relationship with it. Nurses can make use of the results of the present study in order to enhance the quality of healthcare and compatibility with certain situations experienced by every patient. They should be effective in changing the patients’ lives in short run by resorting to the concept of hope. Moreover, nurses should take into consideration this point that patients’ interpretation of chemotherapy should be realistic, so they shit their attention toward other methods of treatment if chemotherapy fails. Furthermore, since the present study was the first one of this type in Iran, it can be adopted as a basis for future studies.
